# Arginine Deiminase Induces Immunogenic Cell Death and Is Enhanced by *N*-acetylcysteine in Murine MC38 Colorectal Cancer Cells and MDA-MB-231 Human Breast Cancer Cells In Vitro

**DOI:** 10.3390/molecules26020511

**Published:** 2021-01-19

**Authors:** Zhiying Huang, Haifeng Hu

**Affiliations:** 1 School of Pharmacy, Shanghai Jiao Tong University, Shanghai 200240, China; decrest@sjtu.edu.cn; 2 Shanghai Institute of Pharmaceutical Industry, China State Institute of Pharmaceutical Industry, Shanghai 201203, China

**Keywords:** arginine deiminase, *N*-acetylcysteine, cell cycle arrest, mitochondrial membrane potential, immunogenic cell death, BMDC phagocytosis

## Abstract

The use of arginine deiminase (ADI) for arginine depletion therapy is an attractive anticancer approach. Combination strategies are needed to overcome the resistance of severe types of cancer cells to this monotherapy. In the current study, we report, for the first time, that the antioxidant N-acetylcysteine (NAC), which has been used in therapeutic practices for several decades, is a potent enhancer for targeted therapy that utilizes arginine deiminase. We demonstrated that pegylated arginine deiminase (ADI-PEG 20) induces apoptosis and G0/G1 phase arrest in murine MC38 colorectal cancer cells; ADI-PEG 20 induces Ca^2+^ overload and decreases the mitochondrial membrane potential in MC38 cells. ADI-PEG 20 induced the most important immunogenic cell death (ICD)-associated feature: cell surface exposure of calreticulin (CRT). The antioxidant NAC enhanced the antitumor activity of ADI-PEG 20 and strengthened its ICD-associated features including the secretion of high mobility group box 1 (HMGB1) and adenosine triphosphate (ATP). In addition, these regimens resulted in phagocytosis of treated MC38 cancer cells by bone marrow-derived dendritic cells (BMDCs). In conclusion, we describe, for the first time, that NAC in combination with ADI-PEG 20 not only possesses unique cytotoxic anticancer properties but also triggers the hallmarks of immunogenic cell death. Hence, ADI-PEG 20 in combination with NAC may represent a promising approach to treat ADI-sensitive tumors while preventing relapse and metastasis.

## 1. Introduction

Arginine, a conditionally or semi-essential amino acid that is involved in several metabolic pathways for humans, plays an orchestral role in the growth of certain tumors. In addition to serving as a building block for protein synthesis, arginine is involved in the regulation of immune responses and tumor metabolism [1,2]. Arginine deprivation has been shown to better selectively kill tumor cells than normal cells [3]. Arginine deiminase (ADI) derived from *Mycoplasma* spp. is the most frequently applied arginine-degrading enzyme in clinical trials. For clinical applications, ADI is usually covalently conjugated with molecules of 20 kDa polyethylene glycol (ADI-PEG 20). This modification greatly enhances ADI’s pharmacokinetic circulatory T_1/2_ of approximately 4 h in blood while reducing its antigenicity [4]. ADI-PEG 20, which hydrolyzes arginine into citrulline and ammonia, is currently being investigated in many clinical trials, such as a phase III trial involving hepatocellular carcinoma (HCC), or combined with conventional chemotherapeutic drugs for the treatment of soft tissue sarcoma [5]. Trials have shown no clear benefit from ADI-PEG 20 monotherapy for patients with HCC. ADI-PEG 20 has been demonstrated to be well tolerated in patients [6,7]. Recently, it was demonstrated that ADI-PEG 20 can modulate the tumor immune microenvironment, thereby enhancing the response to anti-PD-1/PD-L1 in mouse models, with results suggesting a possible synergistic interaction [8]. Therefore, the aim of the current study was to investigate the antitumor and pro-immunogenic properties of ADI-PEG 20 on arginosuccinate synthetase 1 (ASS1)-deficient MC38 and MDA-MB-231 cancer cells in vitro. Due to the main limitations of cross-species-specific immunological incompatibility, human cancer cells cannot be directly investigated for their ability to trigger an adaptive immune response [9], so we chose MC38 for in vitro immune assays.

N-acetylcysteine (NAC), a precursor of reduced glutathione (GSH) in cells, is widely used in clinical therapeutic practices to modulate the intracellular redox state, in addition to its applications in bronchitis, chronic obstructive pulmonary disease (COPD), and chemotherapy-induced toxicity [10,11]. Recently, NAC has also been used as an anticancer agent in vitro and in vivo, either as a stand-alone or as an adjuvant, to reduce cell growth in several types of cancers [12,13,14,15,16].

One novel approach in cancer therapy is to induce immunogenic cell death (ICD), which triggers antitumor immune responses. Most anticancer agents do not kill cancer cells by activating an adaptive immune response. Only a few drugs have the ability to induce an immunogenic modality of ICD: these include chemotherapeutic agents, physical therapy, and oncolytic viruses [17,18,19]. Recent evidence underscores the idea that several therapeutic antibodies [20,21] targeting cell surface-expressed proteins, or some kinase inhibitors [22,23,24], also induce ICD through on-target or off-target effects, suggesting an immune modulation role contributing to their clinical antitumor efficacy as well.

ICD usually involves the cell surface exposure and release of highly immunostimulatory host-derived damage-associated molecular patterns (DAMPs) by dying cancer cells. The extracellular release of high mobility group box 1 (HMGB1) and adenosine triphosphate (ATP) attracts and activates antigen-presenting cells (APCs), and the translocation of calreticulin (CRT) on the surface of dying cancer cells serves as an “eat-me” signal to phagocytes [25,26,27]. Clinical trials with chemotherapy demonstrated the beneficial immunomodulatory effects of ICD induction; more clinical studies are ongoing to investigate the clinical efficacy of bona fide ICD-inducing chemotherapeutic drugs and their correlation with immune biomarkers relevant to disease progression [24,28,29]. It will be crucial to devise highly effective combination regimens that utilize the ability of some treatments to promote ICD [30].

In this study, we propose a combination strategy to enhance the anticancer activity of ADI-PEG 20. Here, we report, for the first time, that ADI-PEG 20 plus NAC decreases cancer cell viability by driving bona fide ICD in vitro. ADI-PEG 20 alone only induced CRT exposure; however, the in vitro phagocytosis assay shows that ADI-PEG 20- but not NAC-treated MC38 cells can be phagocytosed by BMDCs; when combined with NAC, ADI-PEG 20 was capable of inducing the hallmarks of immunogenicity in vitro. Herein, we show that synthetic induction of ICD upon treatment with NAC as an adjuvant may improve the efficacy of ADI-PEG 20 therapy.

## 2. Results

### 2.1. Induction of Apoptosis in Cancer Cells Treated with ADI-PEG 20 and NAC

**Figure 1:**
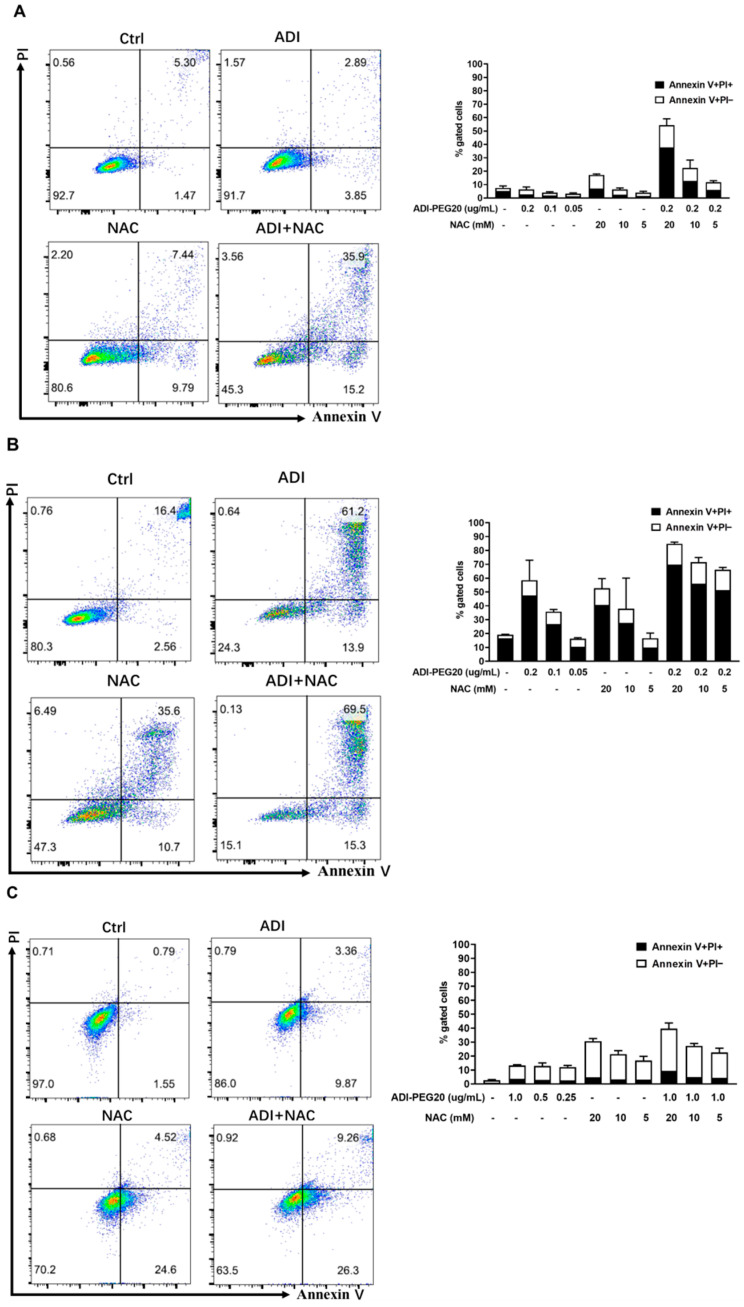

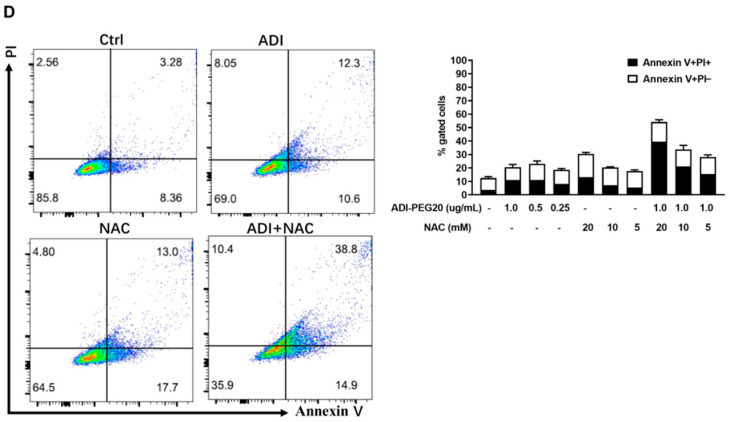
Apoptosis induction by ADI-PEG 20 and NAC. A summary of early (Annexin+/PI−) and late (Annexin+/PI+) apoptotic cell death after 24 h (**A**) and 48 h (**B**) of treatment of MC38 cells and MDA-MB-231 cells for 24 h (**C**) and 48 h (**D**). The *x*-axis represents Annexin V-FITC staining, and the *y*-axis represents PI staining. Each experiment was conducted in triplicate. Ctrl: no treatment control; PI: propidium iodide.

To evaluate whether ADI-PEG 20 or NAC exerts antitumor effects against cancer cells, we firstly performed an annexin-V/propidium iodide (PI) assay to evaluate cell viability in response to ADI-PEG 20/NAC in both MC38 and MDA-MB-231 cells. The dose¬–response results regarding anticancer effects of ADI-PEG 20 and NAC in MC38 and MDA-MB-231 cells are presented in Figure 1. The combination of ADI-PEG 20 (0.2 μg/mL for MC38 and 1 μg/mL for MDA-MB-231 cells, which were selected for further experiments) with an increasing concentration of NAC from 5 to 20 mM was able to enhance the cytotoxic effect in MC38 (Figure 1A,B) and MDA-MB-231(Figure 1C,D) cells; we selected 20 mM NAC for further experiments.

**Figure 2:**
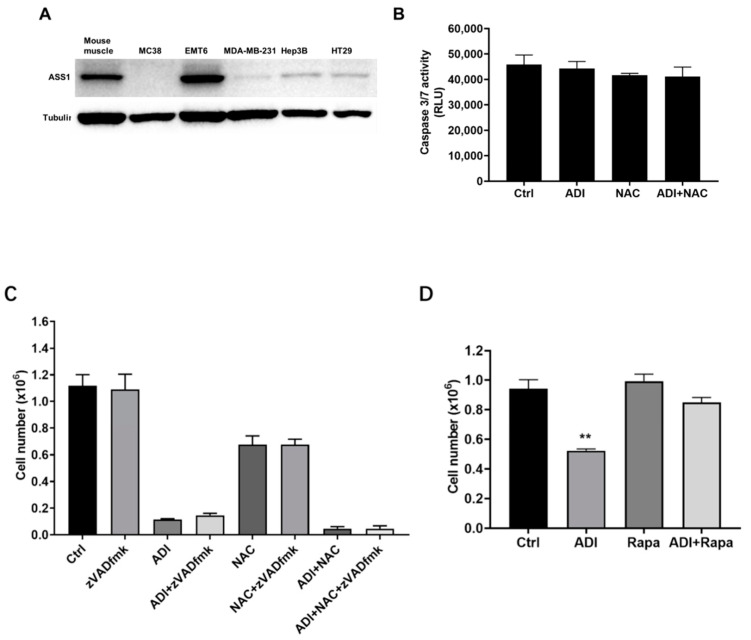
Effect of rapamycin and z-VAD-fmk on ADI-PEG 20-induced cell death in MC38 cells. (**A**) MC38, EMT6, MDA-MB-231, Hep3B, and HT29 cells were examined for ASS1 protein expression by Western blot analysis. Mouse muscle sample was served as ASS1-positive. (**B**) Caspase 3/7 activity of MC38 cells after 24 h of treatment. (**C**) MC38 cells were treated with ADI-PEG 20 (0.2 μg/mL) and NAC with or without z-VAD-fmk (50μM) for 48 h. (**D**) MC38 cells were treated with ADI-PEG 20 (0.025 μg/mL), with or without rapamycin (400 nM) for 48 h, and cell viability was then determined by trypan blue exclusion assay. The data are presented as the mean ± SD from 3 independent experiments. ** *p* < 0.01 (comparison to the control).

To investigate the mechanism responsible for the arginine dependence of MC38 and MDA-MB-231 cells, we analyzed protein expression using Western blot, for expression of the ASS1 protein. The expression of the ASS1 enzyme was absent in MC38 and MDA-MB-231 cell lines, whereas EMT6 cells showed sustained expression of the enzyme (Figure 2A). The time- and dose-dependent MC38 cells’ death in response to ADI-PEG 20 and NAC treatment prompted us to determine whether ADI-PEG 20 and NAC induce caspases, especially caspase-3/7, known to be activated during apoptosis. Cells were treated with ADI-PEG 20 and/or NAC for 24 h, after which caspase-3/7 activity was measured using the Promega Caspase-Glo 3/7 Assay kit. Caspase-3/7 activity was not affected by ADI-PEG 20 and/or NAC treatment (Figure 2B). Caspase-dependent cell death was further investigated using z-VAD-fmk, a pan-caspase inhibitor. z-VAD-fmk did not affect the ratio of dead cells after ADI-PEG 20 treatment (Figure 2C). These results suggest that ADI-PEG 20 and NAC triggers caspase-independent apoptotic cell death in MC38 cells. mTOR signaling controls a diverse set of key cellular processes, such as nutritional status, to modulate eukaryotic cell growth, protein synthesis, and autophagy [31]. In order to assess whether it has a role in ADI-PEG 20-induced cell death, we investigated whether rapamycin (Rapa) could alter cell survival under ADI-PEG 20 treatment. Interestingly, Figure 2D shows that pretreatment of MC38 cells with Rapa at a concentration of 400 nM that did not compromise cell viability significantly rescued cell death induced by ADI-PEG 20 alone. These results suggest that mTOR signaling has an important role in the response of MC38 cells to ADI-PEG 20 treatment.

### 2.2. ADI-PEG 20 and NAC Inhibited Proliferation Markers and Induced Cell Cycle Arrest

**Figure 3:**
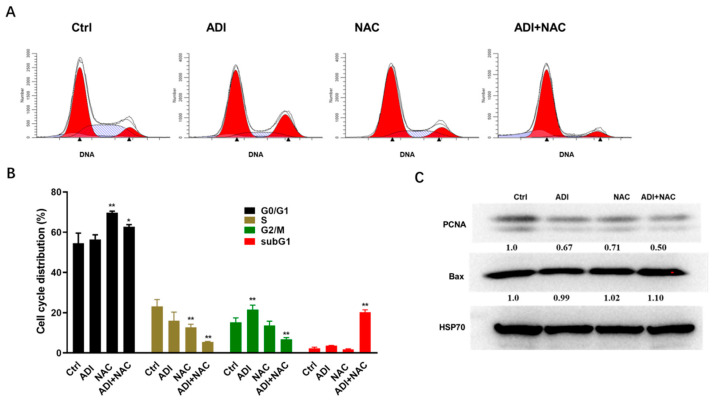
Induction of cell cycle disturbance in MC38 cell lines. (**A**) MC38 cells were treated with ADI-PEG 20 and NAC for 24 h, and then cells were stained with PI to evaluate cell cycle progression. (**B**) The percentages of cells in various cell cycle phases among ADI-PEG 20- and NAC-treated MC38 cells relative to control. Mean values ± SD of three separate experiments performed in triplicate. (**C**) Representative images of immunoblotting analysis of Bax and proliferating cell nuclear antigen (PCNA). * *p* < 0.5, ** *p* < 0.01 (comparison to the control).

To explore the role of cell cycle arrest in the cytotoxic effects of ADI-PEG 20, MC38 cells were stained with propidium iodide (PI), a DNA intercalating agent that can be used to determine the DNA content per cell. As shown in Figure 3A,B, the ADI-PEG 20- and NAC-treated groups had significantly higher percentages of MC38 cells in the G0/G1 phase compared with the control cells. In contrast, the percentages of ADI-PEG 20- and NAC-treated MC38 cells in the S and G2/M phases were decreased correspondingly compared with the control cells. When ADI-PEG 20 was combined with NAC, a significant increase in the SubG1 population was observed in PI experiments (Figure 3B). Proliferating cell nuclear antigen (PCNA) is essential for processive complexation with DNA polymerases by physically tethering the polymerases to DNA. In addition, PCNA acts as a loading platform for proteins involved in cell cycle control and repair [32]. The results of the intracellular Western blot showed that ADI-PEG 20 or NAC alone significantly inhibited PCNA expression in MC38 cells, and combined treatment had additional effects on PCNA expression. Bax is a member of the Bcl-2 family and a core regulator of apoptosis, able to form oligomers and puncture the mitochondrial outer membrane, resulting in apoptosis [33]. As shown in Figure 3C, the administration of ADI-PEG 20 combined with NAC did not alter the Bax protein level in MC38 cells.

### 2.3. Mitochondrial Dysfunction may be Involved in ADI-PEG 20- and NAC-Induced Apoptosis

**Figure 4:**
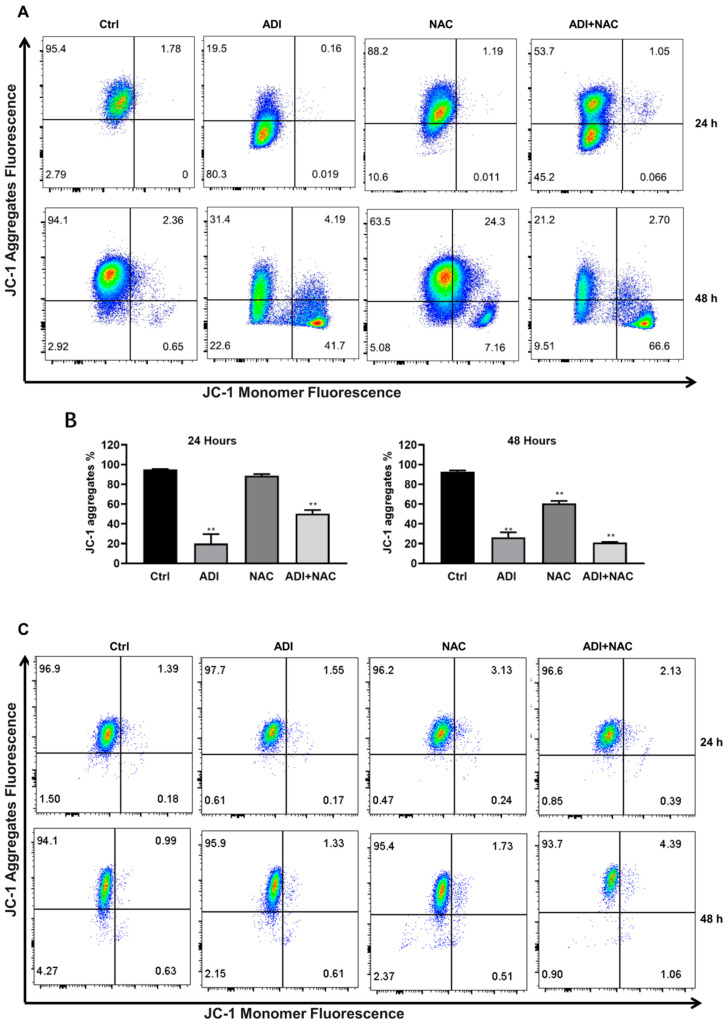

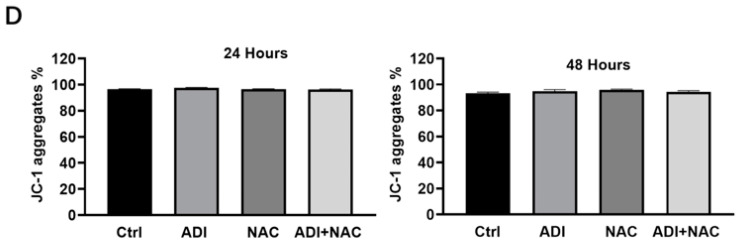
Effects of ADI and NAC on mitochondrial membrane potential. (**A**) Effects of ADI-PEG 20 and NAC at different time courses (24, 48 h) in MC38 cell mitochondrial membrane potential. (**B**) Bar graph representing the percentages of JC-1 aggregates of the JC-1-stained MC38 cells. (**C**) Effects of ADI-PEG 20 and NAC at different time courses (24, 48 h) in MDA-MB-231 cell mitochondrial membrane potential. (**D**) Bar graph representing the percentages of JC-1 aggregates of the JC-1-stained MDA-MB-231 cells. Mean values ± SD of three separate experiments performed in triplicate. ** *p* < 0.01 (comparison to the control).

**Figure 5:**
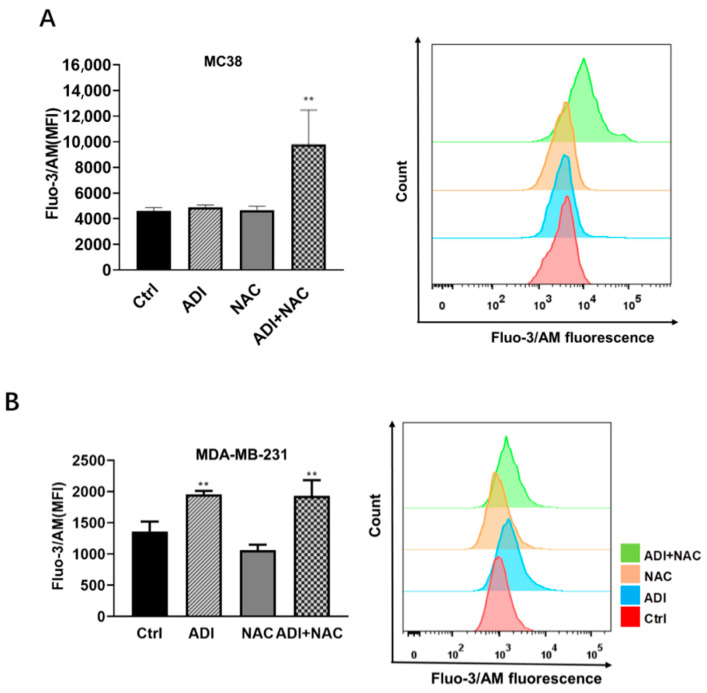
Changes in intracellular Ca^2+^ concentrations of (**A**) MC38 and (**B**) MDA-MB-231 cells by ADI and NAC treatment for 48 h were assayed by Fluo-3/AM staining. MFI: mean fluorescence intensity. ** *p* < 0.01 (comparison to the control).

It has been reported that arginine deprivation induces mitochondrial dysfunction [34], and the current study demonstrates that NAC enhances ADI-PEG 20-induced apoptosis. These findings led to the hypothesis that NAC may influence ADI-PEG 20-induced apoptosis by regulating mitochondrial dysfunction. To further understand the mechanisms of enhanced cytotoxicity induced by the combination of ADI-PEG 20 and NAC, we examined the effects on mitochondrial membrane depolarization. For this reason, we used JC-1 staining to examine the changes in the mitochondrial membrane potential (MMP) in MC38 cells after treatment with ADI-PEG 20 or NAC for 24 and 48 h. Interestingly, we found that the MMP was clearly decreased in MC38 cells treated with ADI-PEG 20 for 24h but partially rescued by NAC (Figure 4A,B). After treatment with ADI-PEG 20 and NAC for 48 h, the MMP of MC38 cells was significantly decreased. ADI-PEG 20 and NAC treatments have no effect on MDA-MB-231 cells (Figure 4C,D) during 48 h of treatment. Since intracellular Ca^2+^ may contribute to cancer cell apoptosis [35], we further assayed the intracellular Ca^2+^ by Fluo-3/AM staining and FACS analysis; we found that the combination treatment increased the intracellular Ca^2+^ concentration in MC38 (Figure 5A) and MDA-MB-231 (Figure 5B) cells. These results suggest that the synergistic effect of ADI-PEG 20 and NAC in inducing apoptosis may involve mitochondrial membrane depolarization and Ca^2+^ homeostasis in MC38 cells.

### 2.4. ADI-PEG 20 Combined with NAC to Promote Immunogenic Cancer Cell Death

**Figure 6:**
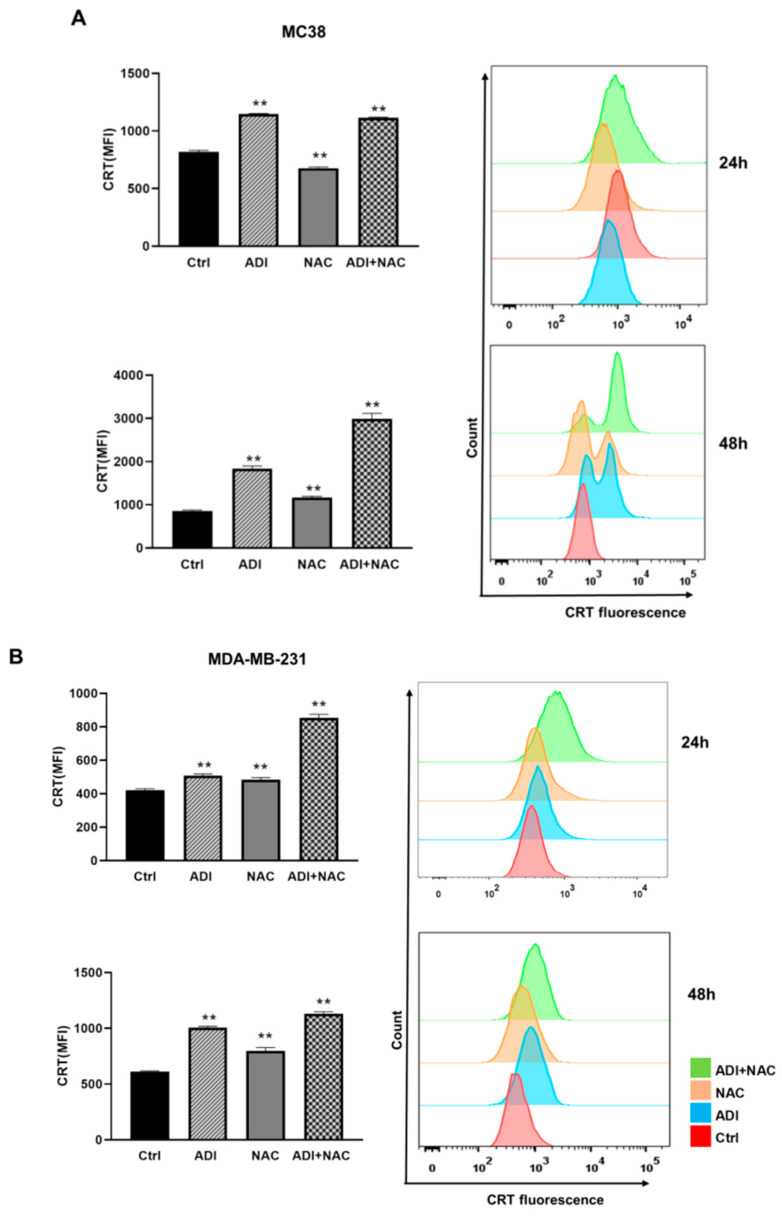

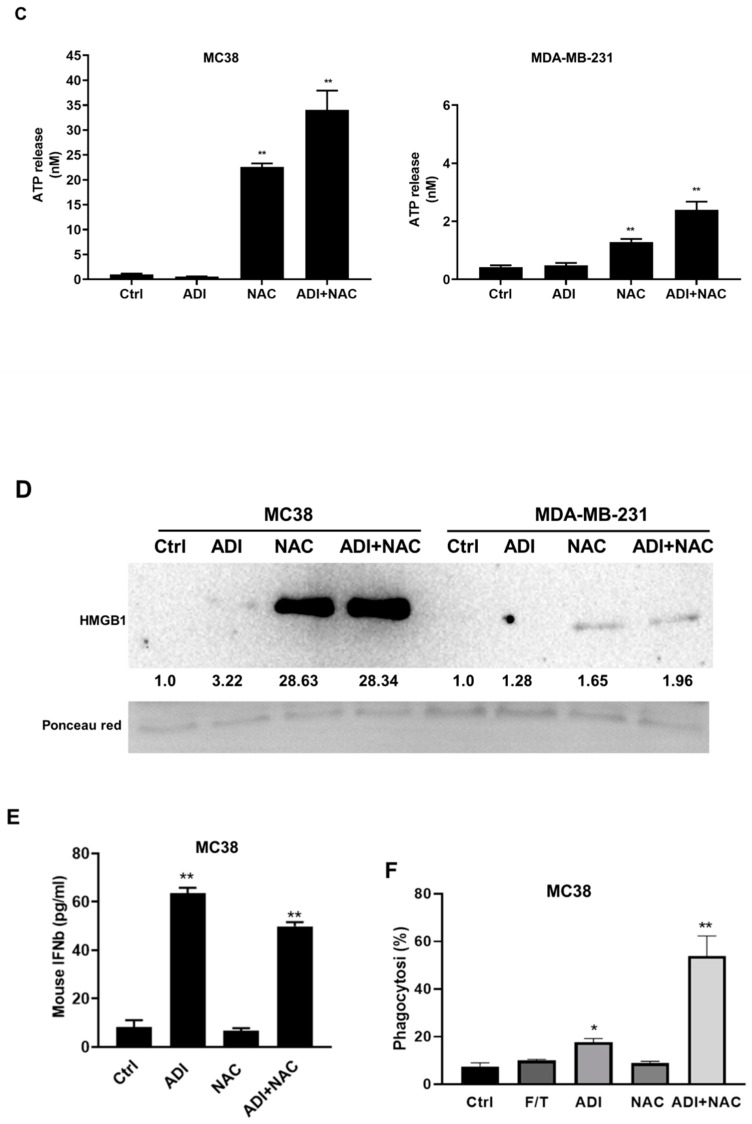
ADI and NAC treatment induced immunogenic cell death (ICD) and promoted bone marrow-derived dendritic cell (BMDC) phagocytosis. (**A**) Assessment of cell surface CRT in MC38 cells treated with ADI-PEG 20 and NAC for 24 and 48 h. (**B**) Assessment of cell surface CRT in MDA-MB-231 cells treated with ADI-PEG 20 and NAC for 24 and 48 h. (**C**) The concentration of extracellular ATP in MC38 and MDA-MB-231 cells was measured by bioluminescence ATP reporter assay. (**D**) HMGB1 release into the cell culture supernatant at 48 h from MC38 and MDA-MB-231 cells treated with ADI-PEG 20 and NAC was detected by Western blot. Ponceau red staining was used as a protein-loading control. (**E**) Mouse IFNb release in MC38 cells was assayed using a mouse IFNb ELISA kit (R&D systems) after 48 h of treatment. (**F**) Phagocytosis of MC38 cells after 24 h treatment by mouse BMDCs was assessed by flow cytometry. F/T: frozen/thawed killed cells. * *p* < 0.5, ** *p* < 0.01 (comparison to the control).

Calreticulin exposure on the cell surface has been identified as a crucial feature in determining the immunogenicity of cancer cell death [36]. In our study, to evaluate the potential of ADI-PEG 20 and NAC to enhance antitumor efficacy or induce ICD, we determined the cell surface translocation of CRT. MC38 and MDA-MB-231 cells were treated with ADI-PEG 20 and/or NAC for 48 h; the cells were then harvested and stained with anti-CRT antibody for FACS. As shown in Figure 6A,B, ADI-PEG 20 or NAC alone caused significant expression of CRT on the cell surface (indicated as the mean fluorescence intensity). Interestingly, treatment with ADI-PEG 20 in combination with NAC resulted in a remarkable increase in CRT exposure on the cell surface as compared with that on the cells treated with each alone.

The ATP concentration of the cell culture medium increased significantly upon NAC treatment, but not with ADI-PEG 20 alone. Treatment with an ADI-PEG 20/NAC combination induced the highest release of ATP following treatment for 48 h (Figure 6C). As shown in Figure 6D, ADI-PEG 20 plus NAC treatment stimulated a significant increase in the amount of extracellular HMGB1 when compared with the control in MC38 cells. We observed only a slight but significant difference in the amount of HMGB1 released between control MC38 cells and MC38 cells treated with ADI-PEG 20 alone. The HMGB1 release in MDA-MB-231 cells was not comparable to that in MC38 cells. Type I interferons (IFNs) such as IFNβ are cytokines with promising potential in antitumor therapy. It has been reported that type I IFNs constitute central coordinators of tumor immune system interactions, not only antagonizing viruses but also antagonizing cancer cells [37]. As shown in Figure 6E, ADI-PEG 20 stimulated IFNβ release by cultured MC38 cells after 48 h. NAC alone had no effect on the IFNβ level, while NAC combined with ADI-PEG 20 maintained stimulation of IFNβ secretion.

To assess whether ADI-PEG 20 and NAC treatment rendered the dying cells more susceptible to phagocytosis by bone marrow-derived dendritic cells (BMDCs), we incubated MC38 cells, either treated or not treated with ADI-PEG 20 and NAC, with BMDCs. FACS analysis showed a strong interaction between BMDCs and ADI-PEG 20/NAC-treated MC38 cells after 2 h (Figure 6F). Notably, we found that MC38 cells treated with ADI-PEG 20 (but not NAC) alone were phagocytosed better than untreated or frozen/thawed-treated cells, although not as efficiently as ADI-PEG 20/NAC-treated cells.

## 3. Discussion

Although arginine deprivation is considered a selective and promising anticancer therapy, it also has some limitations. The first limitation on the effectiveness of this monotherapy is the requirement of arginosuccinate synthetase 1 (ASS1) deficiency in the targeted cancers [38,39,40]. Another limitation is the unpredictable responses of individual patients to ADI treatment in clinical trials based on the baseline ASS1 levels [6,7]. Thus, there is a need to improve the efficacy of ADI-based combination therapy regimens. Recently, it was demonstrated that ADI-PEG 20 can modulate the tumor immune microenvironment, thereby enhancing the response to anti-PD-1/PD-L1 in mouse models, with results suggesting a possible synergistic interaction [8]. Therefore, the aim of current study was to investigate the antitumor and pro-immunogenic properties of ADI-PEG 20 on ASS1-deficient MC38 and MDA-MB-231 cancer cells (Figure 2A) in vitro. Due to the main limitations of cross-species-specific immunological incompatibility, human cancer cells cannot be directly investigated for their ability to trigger an adaptive immune response [9], so we chose MC38 for in vitro immune assays.

The results of the current study show that the viability of MC38 cells was significantly inhibited when cultured in the presence of ADI-PEG 20 and NAC. Interestingly, these cell death changes occurred more quickly under the combination treatment than under each alone over a period of 24 h compared to 48 h. NAC, known to be an antioxidant or reducing agent, could attenuate cancer cell proliferation, migration, and invasion [41]. In apoptosis, caspase-3 is the major effector [42]. The current study indicated that ADI-PEG 20 alone or combined with NAC did not alter caspase-3/7 activity in the MC38 and MDA-MB-231 cell lines. Similar results have been obtained with ADI-PEG 20 in prostate cancer cell lines [43]. That means that phosphatidylserine migration may contribute to the apoptosis in this study. Mechanistic target of rapamycin (mTOR) is a serine/threonine protein kinase that plays a key part in cellular growth and metabolism. mTOR integrates signaling about the availability of nutrients and energy to coordinate the cellular and organismal physiology [31]. Therefore, we investigated whether the mTOR pathway underwent ADI-PEG 20-induced cell death in our cellular system and found that this was the case (Figure 2D). Treatment with rapamycin (400 nM, an inhibitor of mTORC1) and ADI-PEG 20 significantly increased the viability of MC38 cells (Figure 2D) compared with ADI-PEG 20 treatment alone. Previous studies have also shown that ADI-PEG 20 treatment may induce autophagy as a protective early event, and inhibition of autophagy may enhance the therapeutic efficacy of ADI-PEG 20 treatment [43] Therefore, mTOR may play an important role in ADI-PEG 20-induced cell death, possibly through participating in the regulation of autophagy, although the precise underlying mechanism requires further investigation. In MC38 cells, cell death induced by ADI-PEG 20 as a single agent or in combination with NAC (with which the combination effect was greatest) may involve the mitochondria-dependent apoptotic pathway, mediated by mitochondrial membrane depolarization, and Ca^2+^ elevation.

We found that NAC can improve the induction of apoptosis and G0/G1 cell cycle arrest. Furthermore, ADI-PEG 20 may block PCNA-associated proliferation signaling to exhibit antitumor effects. Thus, the present study offers a new combination treatment strategy for cancers involving arginine depletion. The precise mechanism underlying ADI-PEG 20- and NAC-induced PCNA downregulation needs further investigation in the future.

It has been disclosed that several chemotherapeutic drugs could induce ICD in some cancer cells [44]. Dying cancer cells can release specific molecules known as DAMPs, which trigger a tumor-specific immune response. The hallmarks of ICD include at least three independent events: (1) early cell surface translocation of CRT on dying cells, (2) extracellular release of ATP, and (3) release of HMGB1 and IFNb in dying cancer cells [9].

The results of our study show, for the first time, that cell surface CRT was significantly increased in MC38 cells after exposure to ADI-PEG 20. We also observed a significant enhancement in HMGB1, ATP, and IFNb secretion after treatment of the MC38 cells with ADI-PEG 20, suggesting that these cells were undergoing ICD. Moreover, we observed phagocytosis of tumor cells with ADI-PEG 20 treatment, but not NAC; NAC could enhance the phagocytosis of dying tumor cells by BMDCs.

The current findings have important clinical implications as an increasing number of immune-oncology therapies are under investigation in preclinical and clinical settings. More recent data have shown that NAC is able to promote the generation of stem-like memory T cells in vitro [45]. NAC stimulated extensive proliferation of T cells in the range of 10–20 mM, while becoming immunosuppressive at higher concentrations [46]. In the current study, we used an NAC concentration of no more than 20 mM; this concentration not only induced tumor cell death, but may also promote memory T cell proliferation. Recently, it was demonstrated that the ADI-PEG 20 enhances the response to anti-PD-1/PD-L1 in mouse models, with results suggesting a possible synergistic interaction [8]. Our current study shows that the efficacy of ADI-PEG 20 was enhanced in combination with NAC, suggesting that the combination of ADI-PEG 20, NAC, and anti-PD-1/PD-L1 blockade may be a promising therapeutic approach for the treatment of cancers.

## 4. Materials and Methods

### 4.1. Compounds and Reagents

ADI-PEG 20 was prepared in our lab; the specific activity was about 12 unit/mg. N-acetylcysteine was purchased from Sigma. NAC stock (100 mM) was prepared freshly in complement culture media and adjusted to pH 7.4 by adding 2.5 mM NaOH.

### 4.2. Cell Lines and Cell Culture Conditions

MC38 murine colon cancer cells were cultured in Dulbecco’s modified Eagle’s medium containing 1% GlutaMAX™ and grown at 37 °C in a humidified 5% CO_2_/air atmosphere. MDA-MB-231 human breast cancer cells were maintained in Leibovitz L-15 (L-15) medium and grown at 37 °C in a humidified air atmosphere. All culture media were supplemented with heat-inactivated FBS (10%) and penicillin/streptomycin (1%). Media and supplements for cells were obtained from Invitrogen (California, CA, USA).

### 4.3. Assay of Cell Death

Cell death was evaluated using the Annexin V, FITC Apoptosis Detection Kit (Dojindo, Kummoto, Japan). Briefly, 2 × 10^5^ cells were collected, washed with PBS, and resuspended in 1× binding buffer containing 5μL FITC-conjugated AnnexinV and 5 μL PI, following the manufacturer’s instructions, followed by acquisition by flow cytometry.

### 4.4. Caspase Activity Assay

Quantification of the relative caspase-3/7 activity in condition-treated cells was performed using Caspase-Glo 3/7 Assay kits (Promega, Madison, WI, USA). Five thousand cells were seeded in 100 μL of medium into 96-well plates. Twenty-four hours after seeding, cells were treated with the indicated condition for another 24 h. Caspase-Glo 3/7 reagent was added to the wells and incubated at room temperature for 30 min. The luminescence signal was measured using a multifunction microplate reader (Molecular Devices, San Jose, CA, USA).

### 4.5. Cell Cycle Analysis

MC38 cells were treated with the desired condition in complete medium for 24 h. At the end of the treatment time, cells were detached with trypsin–EDTA followed by the protocol for cell cycle analysis as described earlier [47]. Briefly, 0.5 × 10^6^ cells were fixed in 70% ethanol overnight at 4 °C. Then, cells were incubated with 0.5 mL of propidium iodide (PI)/RNase A solution (Beyotime, Shanghai, China) for 1 h at 4 °C in the dark. The cell cycle distribution was then analyzed by flow cytometry using a flow cytometer (BD Biosciences, San Jose, CA, USA).

### 4.6. Measurement of Mitochondrial Membrane Potential

The mitochondrial membrane potential (MMP) was assayed using the lipophilic cationic probe JC-1 according to a previously described procedure [48]. Briefly, cells were detached and incubated with 2 μg/mL JC-1 for 15 min at 37 °C, then rinsed twice and immediately analyzed using an LSRFortessa Flow cytometer (BD biosciences, San Jose, CA, USA). Stained cells shifting from red to green fluorescence indicate the number of cells exhibiting mitochondrial depolarization.

### 4.7. Measurement of Intracellular Ca^2+^ Concentration

The cellular Ca^2+^ level was assessed using the fluorescent probe Fluo-3/AM. Cells were plated on a 6-well plate the day before treatment; after treatment with the indicated condition, 2 μM Fluo-3/AM was added to the culture medium for 30 min. Then, cells were harvested and resuspended in FACS buffer. The fluorescence intensity was analyzed at the indicated time intervals using an LSRFortessa Flow cytometer (BD biosciences, San Jose, CA, USA).

### 4.8. Western Blotting

Cell lysates were prepared by resuspending 2.0 × 10^6^ cells in 200 μL of RIPA lysis buffer containing proteinase inhibitors, disrupting by sonication, and extracting using an ice-water bath. The proteins were electrotransferred to PVDF Immobilon-P membranes (Millipore, Burlington, MA, USA). Specific protein bands were detected by primary antibodies against PCNA, Bax, tublin, and HSP70 (Cell Signaling Technology, Danvers, MA, USA). After 60 min of incubation with HRP-conjugated secondary antibodies at room temperature, immunoreactive proteins were visualized using an enhanced chemiluminescence system. In the case of detecting HMGB1 release to the culture medium, cell culture supernatants were transferred to a clean tube and centrifuged for 10 min at 300× *g*; then, 20 μL of supernatants was mixed with SDS-PAGE loading buffer for further procedures. Specific protein bands were detected by primary antibodies against HMGB1 (Sigma, St. Louis, MO, USA). Reversible Ponceau staining of membranes was performed to check the equal loading of supernatants.

### 4.9. Detection of Cell Surface-Translocated CRT

Cells were trypsinized and washed twice with FACS buffer, and then incubated with anti-CRT antibody (Abcam, Cambridge, MA, USA) at 1:200 dilution in 100 μL FACS buffer at 4 °C for 1 h. Then, cells were washed and stained with FITC-conjugated secondary antibody (Invitrogen, Carlsbad, CA, USA) for 30 min, followed by staining with propidium iodide (PI, Invitrogen) and acquisition by flow cytometry, and the fluorescent intensity of stained live cells was gated on propidium iodide (PI)-negative cells.

### 4.10. ATP Detection

ATP levels in culture medium were detected using the ENLITEN ATP Assay kit (Promega, Madison, WI, USA), following the manufacturer’s instructions, and light intensity was measured using a microplate reader (Molecular Devices, San Jose, CA, USA).

### 4.11. Detection of Mouse IFNb

Supernatants from MC38 cells maintained in control medium or exposed to conditional medium as described above were collected. Quantification of IFNb release was determined using an ELISA-based IFNb detection kit (R&D, Minneapolis, MN, USA).

### 4.12. In Vitro Phagocytosis Assay

To evaluate mouse bone marrow-derived dendritic cells (BMDCs) phagocytosis in vitro, mouse bone marrow cells were isolated from C57BL/6 mice and cultured in complete RPMI 1640 medium, supplemented with mouse recombinant macrophage colony stimulating factor (GMCSF, 20 ng/mL, Sinobiological, Beijing, China) and IL-4 (10 ng/mL, Sinobiological) for 5 days to generate immature BMDCs. MC38 cells (1.0 × 10^6^ cells/mL) were labeled with a nonpermeable dye ATTO 647N NHS ester (50 ng/mL, Sigma) and seeded in 24-well plates for treatment. The day after, cells were treated with ADI plus NAC or single components for 24 h. MC38 cells (0.1 × 10^6^ cells/well) were co-cultured with BMDCs at a 1:1 ratio for 4 h. Cells were then stained with PE-Cy7-labeled anti-mouse CD11c antibody (BD Biosciences, San Jose, CA, USA) at 4 °C for 40 min. Cells were recovered and washed twice, and then analyzed using a flow cytometer (BD Biosciences, San Jose, CA, USA). BMDCs that phagocytosed MC38 cells were ATTO 647N- and mCD11c (the mouse dendritic cell marker)-double-positive.

### 4.13. Statistical Analyses

Results are expressed as the mean ± SD. Statistical analyses were carried out using the Graphpad Prism software package. Statistical differences between more than two groups were determined using one-way ANOVA. Pairwise comparisons were performed using post hoc Dunnett’s multiple comparison tests. A P value of less than 0.05 was considered to indicate statistical significance.

## Data Availability

The data presented in this study are available on request from the corresponding author.

## Sample Availability

Samples of the compounds are not available.
